# Machine learning models using non-linear techniques improve the prediction of resting energy expenditure in individuals receiving hemodialysis

**DOI:** 10.1080/07853890.2023.2238182

**Published:** 2023-07-28

**Authors:** Alainn Bailey, Mohamed Eltawil, Suril Gohel, Laura Byham-Gray

**Affiliations:** a Department of Clinical and Preventive Nutrition Sciences, School of Health Professions, Rutgers University, New Brunswick, NJ, USA; b Department of Health Informatics, School of Health Professions, Rutgers University, New Brunswick, NJ, USA

**Keywords:** Resting energy expenditure, machine learning, chronic kidney disease, predictive equation, hemodialysis, dialysis, lean body mass

## Abstract

**Purpose:**

Approximately 700,000 people in the USA have chronic kidney disease requiring dialysis. Protein-energy wasting (PEW), a condition of advanced catabolism, contributes to three-year survival rates of 50%. PEW occurs at all levels of Body Mass Index (BMI) but is devastating for those people at the extremes. Treatment for PEW depends on an accurate understanding of energy expenditure. Previous research established that current methods of identifying PEW and assessing adequate treatments are imprecise. This includes disease-specific equations for estimated resting energy expenditure (eREE). In this study, we applied machine learning (ML) modelling techniques to a clinical database of dialysis patients. We assessed the precision of the ML algorithms relative to the best-performing traditional equation, the MHDE.

**Methods:**

This was a secondary analysis of the Rutgers Nutrition and Kidney Database. To build the ML models we divided the population into test and validation sets. Eleven ML models were run and optimized, with the best three selected by the lowest root mean squared error (RMSE) from measured REE. Values for eREE were generated for each ML model and for the MHDE. We compared precision using Bland-Altman plots.

**Results:**

Individuals were 41.4% female and 82.0% African American. The mean age was 56.4 ± 11.1 years, and the median BMI was 28.8 (IQR = 24.8 − 34.0) kg/m^2^. The best ML models were SVR, Linear Regression and Elastic net with RMSE of 103.6 kcal, 119.0 kcal and 121.1 kcal respectively. The SVR demonstrated the greatest precision, with 91.2% of values falling within acceptable limits. This compared to 47.1% for the MHDE. The models using non-linear techniques were precise across extremes of BMI.

**Conclusion:**

ML improves precision in calculating eREE for dialysis patients, including those most vulnerable for PEW. Further development for clinical use is a priority.

## KEY MESSAGES

Potentially impacting millions of patients worldwide, our continuing goal is to understand energy expenditure (EE) across the spectrum of CKD (stages 1–5) in adults and children being treated with dialysis or transplantation, with the intent of providing tools for the health professional that will improve the delivery of quality care.

In past research, we have identified and focused on disease-specific variables which account for 60% of the variance in predicting EE in individuals receiving dialysis, but many questions remain unanswered.

Our hypotheses are that (1) there are determinants of EE specific to CKD and, (2) predicting EE for individuals may be greatly advanced using sophisticated models that combine these determinants. In this study, we applied machine learning (ML) with linear and non-linear techniques to our existing dataset. The best models demonstrated improved precision in predicting EE for all individuals in the validation group.

## Introduction

There are close to 700,000 individuals in the United States with stage 5 chronic kidney disease (CKD) receiving dialysis treatment [1]. Despite ongoing improvements, three-year survival rates for people receiving dialysis are approximately only 50% [1,2]. There are many contributing factors to such poor health outcomes. As the disease progresses, suboptimal nutritional status, inflammation, concomitant hyper-catabolism and metabolic aberrations lead to protein-energy wasting (PEW), which independently exacerbates adverse health sequelae [3–9].

Estimating an individual’s resting energy expenditure (REE) with precision is vital for promoting energy-balance especially among those diagnosed with PEW. It could also provide a cost-effective survival benefit for many people worldwide by targeting the appropriate nutritional and medical interventions [6–12]. However, current clinical methods to assess REE for these individuals have either proven too expensive, such as measured REE (mREE) by indirect calorimetry (IC) or are insufficiently precise to account for specific disease and metabolic circumstances [13,14]. This includes existing predictive energy equations (EEs), such as the Harris-Benedict [15] or Mifflin St Jeor [16] equations which use linear regression algorithms and basic clinical variables such as weight and height [15–27].

Previous investigators have shown that disease-specific variables, such as inflammation, blood glucose levels and renal biomarkers are predictive of REE in individuals receiving dialysis [21,23].

To date, dialysis-specific EEs have improved predictive accuracy by achieving around 60% precision (± 10% of zero difference from mREE) when tested in samples of people with similar characteristics to the development group [20–25]. However, our group has demonstrated that disease-specific EEs may not perform well when transferred to different geographical or demographic samples, or to those people with outlying characteristics, such as very low or high body mass index (BMI) [27]. Arguably, such individuals may be the most vulnerable.

In recent years, more sophisticated machine learning (ML) techniques have been applied to medical diagnostics for hemodialysis patients. These methodologies can improve the prediction of clinical outcomes (such as disease progression and mortality) using common clinical factors [28–32]. ML takes a very different approach to traditional linear regression [33,34]. It engages the variables without preconception to determine algorithmic rules from inherent patterns in that data [34]. This is achieved by randomizing large sets of variables into combinations of linear and non-linear models and can select features that yield complex and nuanced interactions [34,35]. In addition, increases in computational power have tremendously improved the researcher’s ability to process large samples of data in vastly more complex and accurate ways [34,35].

It is our hypothesis that such techniques may also be applied to clinical nutrition. For example, using 114 individuals, Ponce et al. applied ML techniques to predict REE in patients with acute kidney injury (AKI) receiving dialysis [28]. This group used a combination of linear and non-linear regression models including Linear Regression with Stepwise Selection, Linear Regression with Regularization, RPART, Support Vector Machine with Radial Kernel, Generalized Boosting Machine, Extreme Gradient Boosting and Random Forest [28]. The best model (Random Forest) predicted REE with 69% accuracy compared to 24% for the Harris-Benedict Equation [28].

The Rutgers Nutrition and Kidney Database (RNKD) is a large renal database in the USA, containing over 600 clinical and demographic variables gathered for 210 hemodialysis patients over 4 separate studies undertaken between 2012 and 2018 [27]. Using this database, the primary objective of this pilot study was to ascertain if a machine learning approach alone can generate a more precise estimation of REE than previous statistical methods using linear and logistical regression only [20–25]. A secondary purpose was to clinically consider the features selected by the best-performing ML models, to guide the direction of future research.

## Methods

### Study design

This was a secondary analysis of an existing database, the RNKD. The RNKD is an amalgam of four existing studies conducted from 2012 to 2018 [36–39]. The studies were all undertaken in the Northeast and Midwest regions of the United States and only included people receiving maintenance hemodialysis (MHD), 3 times a week for at least 3 months. Sampling for enrollment was conducted by convenience, meaning that individuals in dialysis clinics were asked to volunteer.

Inclusion and exclusion criteria were similar in every study and described in detail by Byham-Gray et al. [21]. Participants were women and men greater than 18 years with stage 5 CKD receiving MHD (conventional method, in-center at a for-profit dialysis unit) 3 times per week for at least 3 months. Exclusion criteria included infective complications or poorly healing wounds, surgical procedures or cardiovascular events within 30 d of enrollment, recreational pharmaceutical usage, frequent ingestion of dietary supplements, a previous diagnosis of heart failure, hepatic disease, or cancer.

### Data collection

All the studies in the RNKD used data-gathering protocols that were alike. The individuals and their medical records provided demographic data. Anthropometric and clinical data were gathered on a day free of dialysis. REE was determined by indirect calorimetry using a metabolic cart (Cosmed Quark RMR®, Rome, Italy). Participants were requested not to exercise vigorously and fast for 12 h before the assessment. If a 12 h fast was not possible then a 4 h fast was requested. The fast was introduced to reduce fluid accumulation and its impact on body weight and composition. IC took place before 12 pm. Participants lay still and awake for at least twenty minutes. The measurement protocol was adopted as previously defined by Olejnik et al. [36] and is fully described in previous studies published by this group [21,23,27].

### Data mining

The RNKD is stored on a password-protected SPSS file on the Rutgers Box platform. After Approval from the Rutgers University Institutional Review Board (Protocol number: Pro2020001656), the dataset was extracted and delivered as a separate SPSS file. All the data were de-identified before further analysis. They were stored on a password-protected laptop and shared only with co-investigators over encrypted email or cloud. As this was a secondary analysis, no further permissions were required.

### Measurement of estimated resting energy expenditure *via* machine learning models

In this study, we provided the largest group of variables possible to the ML models and allowed the models to effectively choose the predictive features for themselves. The RNKD was screened and cleaned for inaccuracies and assessed by two renal dietitians. Variables were excluded if found to be clinically irrelevant to human metabolism, statistically insignificant, or where insufficient values were available to maintain the integrity of the dataset. For the construction of the models, individuals were split between a training set (80% of cases) and a validation set (20% of cases). Although the case selection was random, each set aimed to maintain the BMI distribution of the entire sample so long as the data permitted, as this was pertinent to our ultimate assessment of precision. Preprocessing procedures included assessment of multicollinearity, box and cox transformations, centering and scaling predictors and creating dummy variables constructed based on linear and non-linear combinations of existing variables to assess such combined effects. Where it was deemed appropriate to impute missing variables this was done *via* adding the mean value applied across the variable’s present values.

In total, eleven ML models were developed in the training set using linear and non-linear regression, both internally in the ML regression techniques and in combining variables in each model. The selection included Bayesian Ridge, Elastic Net, Gradient Boosting Regressor, Lasso, Linear Regression, Linear SVR (support vector regression), MLP (multi-layer perception) Regressor, Random Forest, Ridge Regression, SGD (stochastic gradient descent) Regressor and SVR. Given the large number of features present in the dataset (435) vs. the number of patients, a feature selection technique based on optimization of the target root mean squared error (RMSE) and *R* squared (*R*^2^) through progressively eliminating the least impactful feature was developed. Variables were excluded from each model one by one if this lowered the RMSE. The breakpoint was set when marginal exclusion increased RMSE, at which point the number of features was set. The performance of each model was assessed in the validation set using relative analysis of the highest *R*^2^ and the lowest RMSE. We selected the best three models prioritizing RMSE as this reflects the lowest average divergence from mREE in kcal. We then used the best three models to generate estimates of REE in the validation set.

### Measuring estimated resting energy expenditure *via* predictive equation

For this study, the best model of the Maintenance Hemodialysis Equation (including c-reactive protein {CRP}) was used (MHDE-CRP) [27]. The variables used to create values for eREE were age, sex, weight, and CRP. Although not all individuals in the validation group had values for CRP, we assessed that sufficient real values existed, and that imputation of the rest would not substantially alter the explanation of variance. Missing values were imputed using the median CRP for the entire sample (training + validation sets), which reflected the distribution of values within our dialysis population at large.

### Statistical and graphical analyses

No power analysis was undertaken in this study as it was previously included when the MHDE was constructed by this research group [21]. At that time, *n* = 60 was adequate for equation building and *n* = 95 was adequate for validation of the equation. Our latest study included 167 individuals from the same dataset in our ML analysis, hence further investigations on sample size were deemed unnecessary. Furthermore, statistical significance was demonstrated for the relevant findings, indicating that the study utilized a sufficient sample size.

ML models were developed, and analysis performed, using Python (version 8.4.0) and Sci-Kit (version 1.1.1) learn package. Statistical analyses were performed using Statistical Package for Social Sciences (SPSS, IBM Corp., version 27, Armonk NY). If values were found to be normal *via* visual inspection, they were expressed as mean and standard deviation (SD). If not normal, values were stated as median, 25th and 75th percentiles. An intraclass correlation coefficient (ICC) was calculated to analyze the reliability of each equation using a model with a single rater, 2-way mixed-effects and absolute agreement [37]. *Alpha priori* was established at 0.05.

We used a modified Bland–Altman plot to measure the levels of agreement between mREE and eREE from each model [38]. The original Bland-Altman plot graphically assesses agreement between two methods of measurement by examining one method on the Y-axis by comparison with either the true measure on the X-axis or the mean of both measures if the criterion is not known [38]. In this case, we used residual values calculated *via* percentage on the Y-axis and mREE (the criterion measure) on the X-axis. A full description of the method was previously published by this group [27]. Limits of agreement for predictive equations have been established at ± 10% from zero difference from mREE in the nutrition literature [38]. Those limits have been used for validation by Byham-Gray et al. [21,23], Morrow et al. [25] and Bailey et al. [27] when assessing equations for people receiving dialysis [21,23,25,27]. This graphical analysis was applied to each of the best models (and the MHDE) across the complete validation sample for which REE was generated. The analysis was subsequently repeated with the validation set divided into subgroups of BMI. Individuals with a BMI less than 24.9 kg/m^2^, 25–29.9 kg/m^2^, or ≥ 30 kg/m^2^ were categorized as underweight/normal weight, overweight, or obese.

### Clinical and narrative analysis

We categorized the features selected by the best models into groupings to consider their clinical significance. These groupings were, demographic, anthropometric, disease-related, dynamic/clinical, patient-reported and provider-assessed. We then assessed the distribution of features amongst the groups to narratively identify trends that may assist future researchers.

## Results

In total, 167 of the individuals retained sufficient variables for this study. The population was 58.7% male, 82% African American, and 80.2% Non-Hispanic (Table 1). Ages ranged between 21.5 and 80.7 years. The mean age was 56.4 ± 11.4 years (Table 2). The median BMI for the group was 28.8 (IQR = 25.8–34.0) kg/m^2^. 25.7% of individuals were categorized as underweight or normal weight, 35.3% as overweight, and 39.0% as obese. The sample was randomly split into 80% training sample and 20% validation sample while maintaining the BMI stratification constraint. There was no statistical difference in the frequencies of sex, race, ethnicity and BMI between the total and the validation samples.

**Table 1.** Frequency of clinical and demographic characteristics of individuals in the Rutgers Nutrition and Kidney Database (*N* = 167).

	Total		Training set		Validation set	
Variable	*N*	%	*n*	%	*n*	%
Sex						
Male	98	58.7	78	58.6	20	58.8
Female	69	41.4	55	41.3	14	41.2
Ethnicity						
Non-Hispanic	134	80.2	104	78.2	30	88.2
Hispanic	15	9.0	13	9.8	2	5.9
Unknown	18	10.8	16	12.0	2	5.9
Race						
African American	137	82.0	107	80.5	30	88.2
White	30	18.0	26	19.5	4	11.8
BMI						
Underweight/Normal weight (<24.9 kg/m^2^)	43	25.7	34	25.6	9	26.5
Overweight (25–29.9 kg/m^2^)	59	35.3	47	35.3	12	35.3
Obese (≥30 kg/m^2^)	65	39.0	52	39.1	13	38.2

BMI: body mass index; kg/m^2^: kilograms per meter squared.

**Table 2.** Demographic and clinical characteristics among individuals in the Rutgers Nutrition and Kidney Database (*N* = 167).

	Mean ± SD		Median (25–75th percentiles)		Minimum – Maximum	
Variable	Total	Validation Set	Total	Validation Set	Total	Validation Set
Age (years)	56.4 ± 11.4	56.7 ± 11.4	56.7 (49.9**–**64.2)	59.5 (51.6**–**63.4)	22**–**81	30.3**–**76.8
Weight (kg)	85.4 ± 20.2	87.0 ± 23.7	82.1 (71.8**–**97.1)	88.3 (74.3**–**101.2)	45**–**150.8	45.2**–**143.1
Height (cm)	169.4 ± 10.1	170.2 ± 10.8	170.1 (162.0**–**177.0)	174.0 (162.0**–**177.9)	143.9**–**193.6	145.9**–**193.6
BMI (kg/m^2^)	30.1 ± 6.8	29.9 ± 7.8	28.8 (24.8**–**34.0)	28.6 (20.62**–**34.8)	17.0**–**50.8	18.34**–**50.8
CRP (mg/L)	10.5 ± 14.9	10.0 ± 10.2	5.4 (1.3**–**12.0)	6.8 (1.9**–**12.6)	0.1**–**94.0	0.3**–**32.1
Albumin (g/dL)	4.2 ± 0.4	4.2 ± 0.5	4.2 (3.9**–**4.5)	4.2 (3.9**–**4.5)	3.1**–**5.4	3.1**–**5.0

BMI: body mass index; cm: centimeters; CRP: C-reactive protein; g/dL: grams per deciliter; kcal: kilocalories; kg: kilograms; kg/m^2^: kilograms per meter squared; SD: standard deviation.

### Selecting the most accurate machine learning models to predict energy requirements

Eleven ML models were run and optimized within the training set (*N* = 133) to predict REE. Of the full dataset, 43 subjects and 171 variables were omitted due to significant missing data. 188 variables were excluded as they were not deemed clinically relevant, and 11 variables were omitted from modelling as they were not statistically relevant. In total, the optimized models selected 55 features with an individual model range between 8 and 41 features (Table 3).

**Table 3.** Eleven machine learning models for estimating resting energy expenditure ranked by the lowest root mean squared error.

	RMSE		Optimized features (*N* = 65)	Run time
Model	(Kcal)	*R* ^2^	(*n*)	(Seconds)
SVR	103.6	0.918	35	35.0
Linear regression	119.0	0.892	37	19.8
Elastic Net	121.1	0.888	24	25.5
Linear SVR	128.9	0.874	37	22.3
MLP regressor	131.2	0.869	41	1239.0
Bayesian ridge	134.6	0.862	24	22.8
Ridge	136.4	0.858	35	17.7
SGD regressor	139.1	0.853	29	25.6
Lasso	140.9	0.849	26	19.1
Gradient boosting	156.0	0.815	8	170.1
Random forest	161.6	0.801	15	412.4

MLP: multi layer perception; R^2^: correlation coefficient; RMSE: route mean squared error; SGD: stochastic gradient descent; SVR: support vector regression.

The three best models were selected because they exhibited the lowest RMSE (kcal) from mREE. These models were SVR (103.6 kcal), Linear Regression (119.0 kcal) and Elastic Net (121.1 kcal). We then used these models to generate eREE values within the validation set (*N* = 34).

### Measured and predicted energy requirements

The median mREE was 1512.9 kcal/d and ranged from 1079.5 to 2528.1 kcal (Table 4). The median mREE for women (1304.6 kcal/d) was lower than for men (1588.8 kcal/d). The SVR REE had the lowest average prediction of energy requirements, with a median eREE of 1472.8 kcal/d. The Linear Regression REE had the highest average prediction, with a median eREE of 1527.1 kcal/d.

**Table 4.** Measured and estimated resting energy expenditure among individuals in the validation set of the Rutgers Nutrition and Kidney Database (*N* = 34).

Variable	*n*	Mean ± SD	Median (25−75th percentiles)	Minimum − Maximum
mREE (kcal)		1579.4 ± 367.8	1512.9 (1326.8 − 1689.8)	1079.5 − 2528.5
Male	20	1336.2 ± 331.2	1588.8 (1427.3 − 1859.7)	1324.2 − 2528.5
Female	14	1336.2 ± 235.0	1304.6 (1132.0 − 1478.9)	1079.5 − 1873.6
MHDE-CRP REE (kcal)		1512.0 ± 312.3	1493.3 (1330.5 − 1687.7)	967.4 − 2333.9
Male	20	1661.0 ± 229.3	1627.6 (1473.3 − 1786.2)	1346.2 − 2333.9
Female	14	1299.1 ± 296.3	1260.1 (1028.5 − 1492.7)	967.4 − 1969.0
SVR REE (kcal)		1549.6 ± 356.6	1472.8 (1321.2 − 1754.6)	1079.4 − 2624.5
Male	20	1707.5 ± 247.4	1608.0(1463.5 − 1903.5)	1324.5 − 2624.5
Female	14	1324.0 ± 230.6	1309.8 (1416.2 − 1703.4)	1079.4 − 1788.4
Linear REE (kcal)		1552.2 ± 304.6	1527.1 (1347.7 − 1754.6)	1007.0 − 2343.7
Male	20	1682.7 ± 286.6	1605.1 (1523.5 − 1830.5)	1278.3 − 2343.7
Female	14	1365.7 ± 227.6	1348.1(1199.8 − 1535.8)	1007.0 − 1802.9
Elastic REE (kcal)		1548.3 ± 310.5	1501.3 (1339.5 − 1702.0)	1011.0 − 2291.0
Male	20	1704.9 ± 267.2	1601.7 (1495.4 − 1846.6)	1426.6 − 2291.0
Female	14	1324.6 ± 220.9	1408.7 (1133.6 − 1478.1)	1011.0 − 1785.2

Elastic Net REE: The elastic net model for estimating resting energy expenditure; kcal: kilocalories; Linear REE: The linear regression model for estimating resting energy expenditure; MHDE-CRP REE: maintenance hemodialysis C-reactive protein equation for estimating resting energy expenditure; mREE: measured resting energy expenditure; REE: resting energy expenditure; SD: standard deviation; SD: standard deviation, SVR REE: The SVR model for estimating resting energy expenditure.

### Levels of agreement

**Figure 1.**
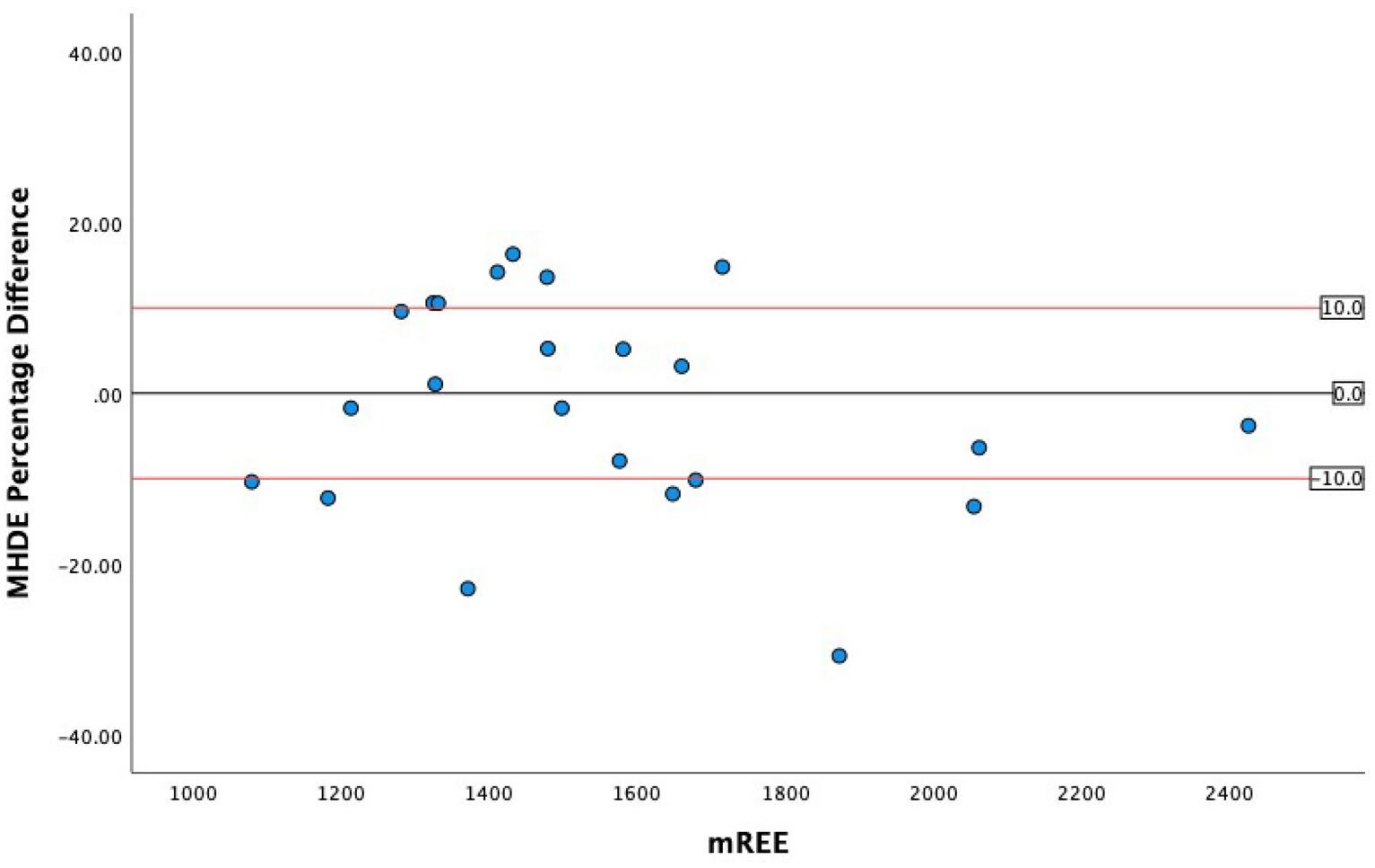
Modified Bland Altman Plot of the Percentage Difference between the MHDE REE and mREE. The black line represents zero difference from mREE. The upper red line represents 10% difference from mREE. The lower red line represents −10% difference from mREE.

**Figure 2.**
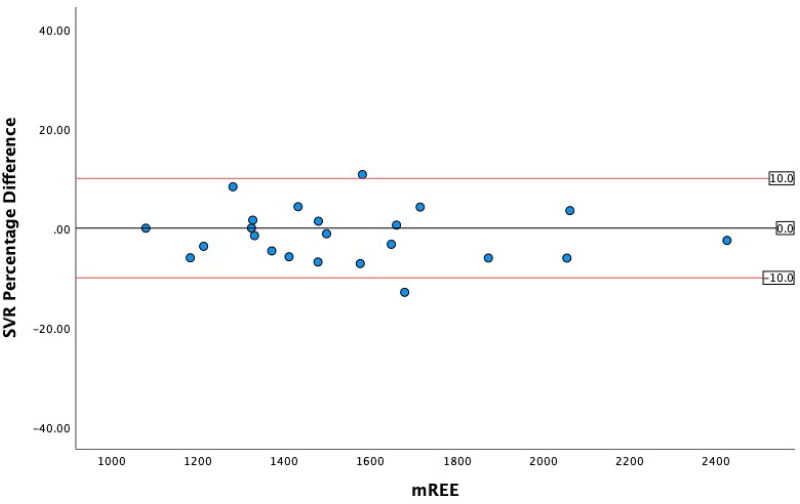
Modified Bland Altman Plot of the Percentage Difference between the SVR REE and mREE. The black line represents zero difference from mREE. The upper red line represents 10% difference from mREE. The lower red line represents −10% difference from mREE.

**Figure 3.**
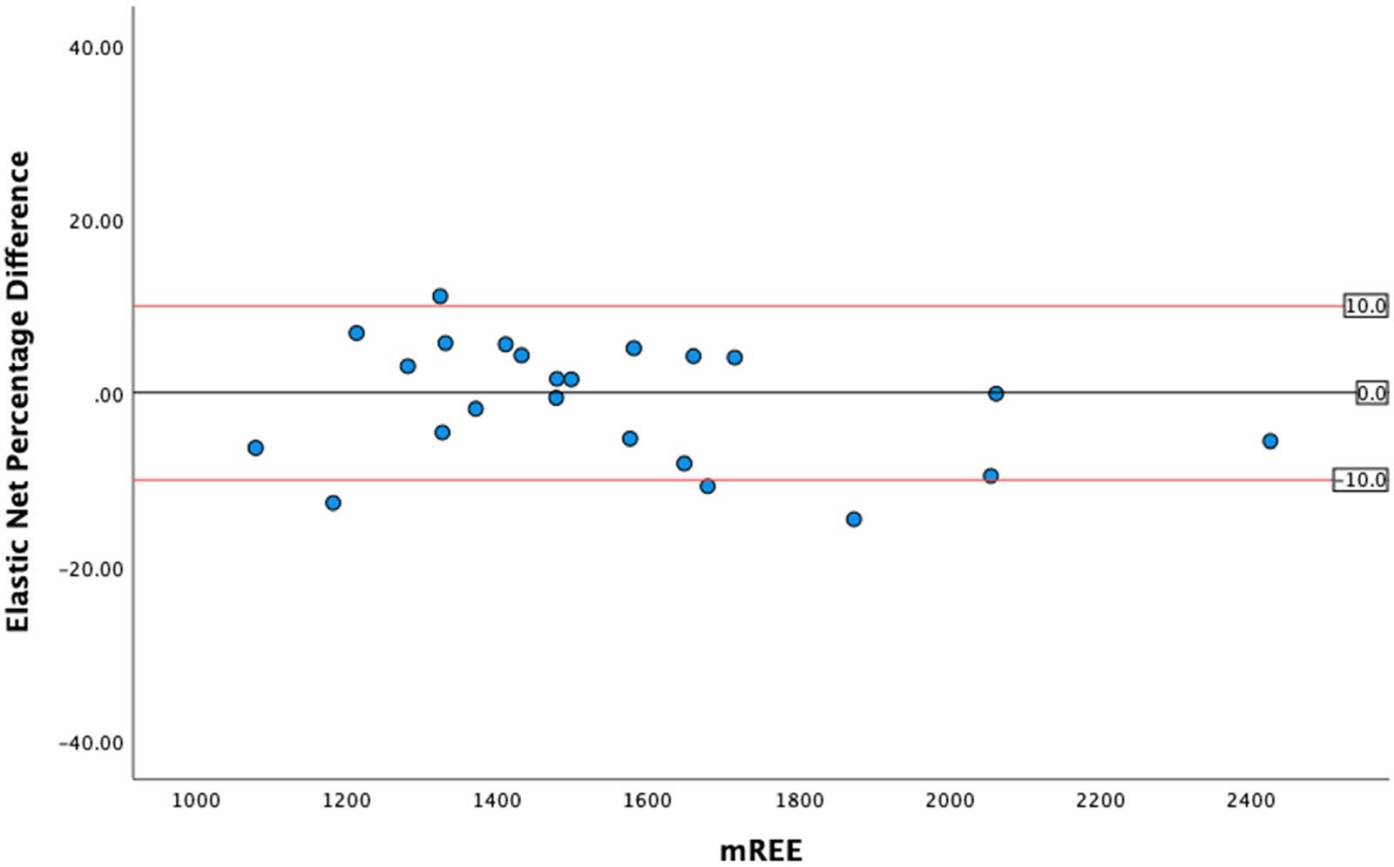
Modified Bland Altman Plot of the Percentage Difference between the linear REE and mREE. The black line represents zero difference from mREE. The upper red line represents 10% difference from mREE. The lower red line represents −10% difference from mREE.

**Figure 4.**
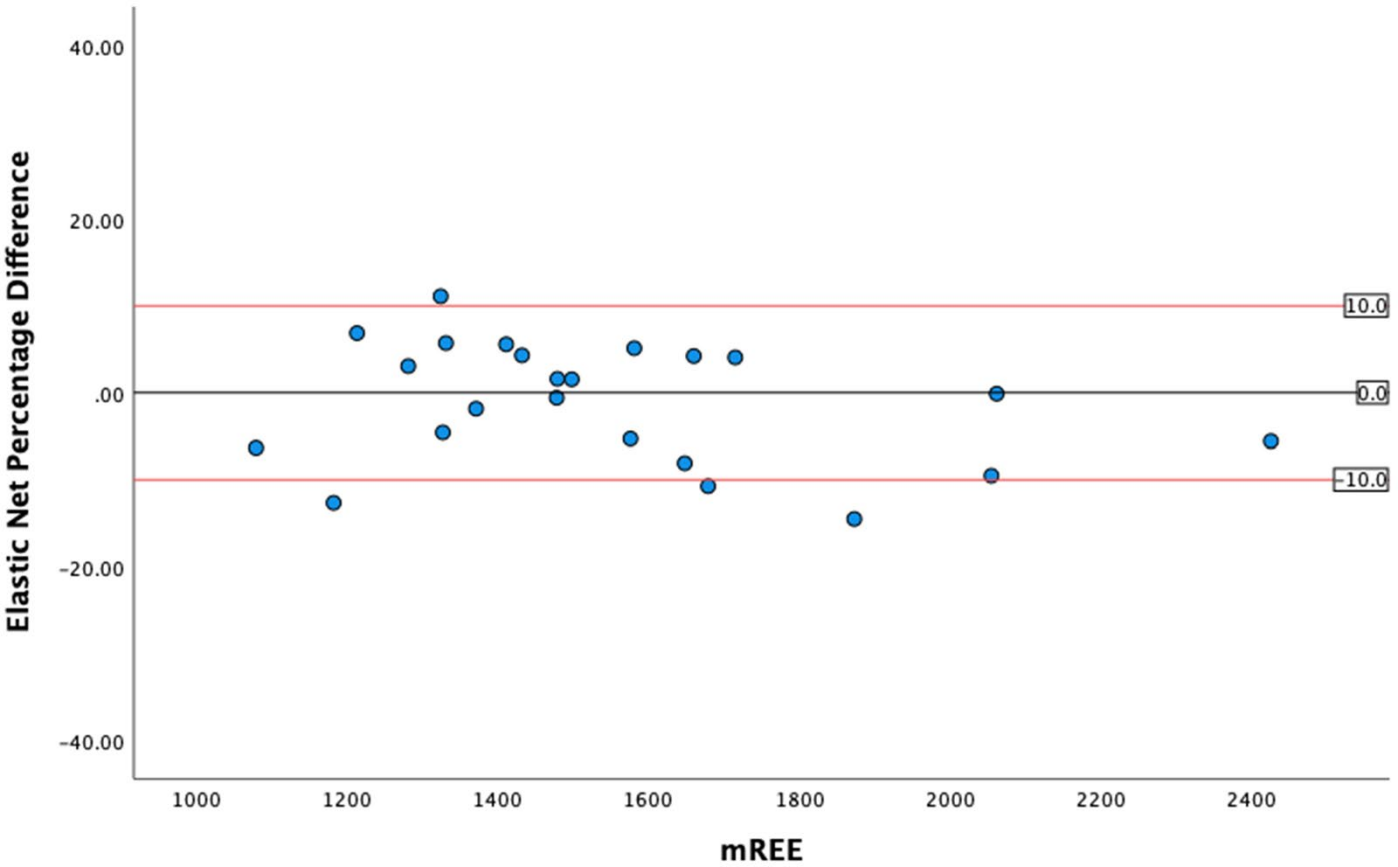
Modified Bland Altman Plot of the Percentage Difference between the Elastic Net REE and mREE. The black line represents zero difference from mREE. The upper red line represents 10% difference from mREE. The lower red line represents −10% difference from mREE.

The greatest level of agreement occurred between mREE and the SVR REE, with 91.2% of values within ± 10% from mREE (Table 5). The other ML models both performed within acceptable limits. However, in this sample, the MHDE REE only predicted 47.1% of values within acceptable limits. ICC analysis demonstrated excellent reliability for the SVR REE and good reliability for the Linear REE and Elastic Net REE and moderate reliability for the MHDE REE. Bland-Altman plots demonstrated that eREE values within acceptable limits were evenly distributed either side of zero difference for the MHDE REE and for all three ML models (Figures 1–4).

**Table 5.** Levels of agreement in resting energy expenditure as derived by indirect calorimetry, Compared to one predictive energy equation and three machine learning models for individuals receiving maintenance hemodialysis (*N* = 34).

Equation/Model	Within ± 10% of mREE *n* (%)	<10% of mREE *n* (%)	> 10% of mREE *n* (%)	Intraclass correlation R	*p* value
MHDE CRP	16 (47.1)	11 (32.4)	7 (20.6)	0.787	<.001
SVR	31 (91.2)	2 (5.9)	1 (2.9)	0.959	<.001
Linear regression	28 (82.4)	5 (14.7)	1 (2.9)	0.938	<.001
Elastic net	27 (79.4)	5 (14.7)	2 (5.9)	0.937	<.001

MHDE CRP: maintenance hemodialysis C-reactive protein equation for resting energy expenditure; mREE: measured resting energy expenditure; REE: resting energy expenditure; SVR: support vector regression.

### Variability of agreement in different categories of BMI

**Figure 5.**
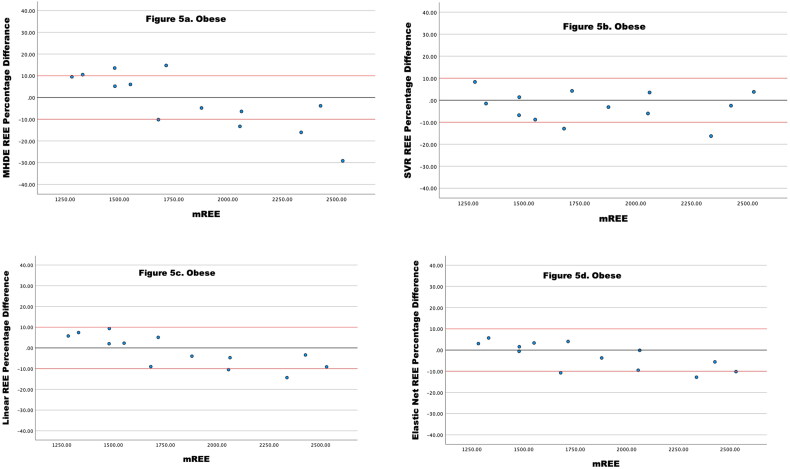
(a–d) Percentage Difference between Four different models of eREE and mREE in people receiving MHD categorized as **obese**. The black lines represent zero difference from mREE. The upper red lines represent a 10% difference from mREE. The lower red lines represent −10% difference from mREE.

For participants with obesity, the SVR REE and Linear REE showed the same levels of accuracy (84.6% within limits) (Table 6) and Elastic Net REE predicted 76.9% of estimates within acceptable limits. For all the ML models, the values outside of limits were underestimated (Figure 5(b–d)). The MHDE REE demonstrated only 46.2% accuracy for obese persons, with inaccurate estimates split evenly between over and underestimation (Figure 5(a)).

**Table 6.** Levels of agreement in resting energy expenditure as derived by indirect calorimetry compared to one predictive energy equation and three machine learning models, stratified by body mass index (*N* = 34).

Equation/Model	Within ± 10% of mREE *n* (%)	<10% of mREE *n* (%)	>10% of mREE *n* (%)
Under/Normal weight			
MHDE CRP	0 (0.0)	8 (89.0)	1 (11.0)
SVR	9 (100.0)	0 (0.0)	0 (0.0)
Linear regression	5 (55.6))	3 (33.4)	1 (11.0)
Elastic net	56 (66.7)	2 (22.2)	1 (11.0)
Overweight			
MHDE CRP	10 (83.3)	0 (0.0)	2 (16.7)
SVR	11 (91.7)	0 (0.0)	1 (8.3)
Linear regression	12 (100.0)	0 (0.0)	0 (0.0)
Elastic net	11 (91.7)	1 (8.3)	0 (0.0)
Obese			
MHDE CRP	6 (46.2)	4 (30.8)	3 (23.1)
SVR	11 (84.6)	2 (15.4)	0 (0.0)
Linear regression	11 (84.6)	2 (15.4)	0 (0.0)
Elastic net	10 (76.9)	3 (23.1)	0 (0.0)

MHDE CRP: maintenance hemodialysis C-reactive protein equation for resting energy expenditure; mREE: measured resting energy expenditure; REE: resting energy expenditure; SVR: support vector regression.

**Figure 6.**
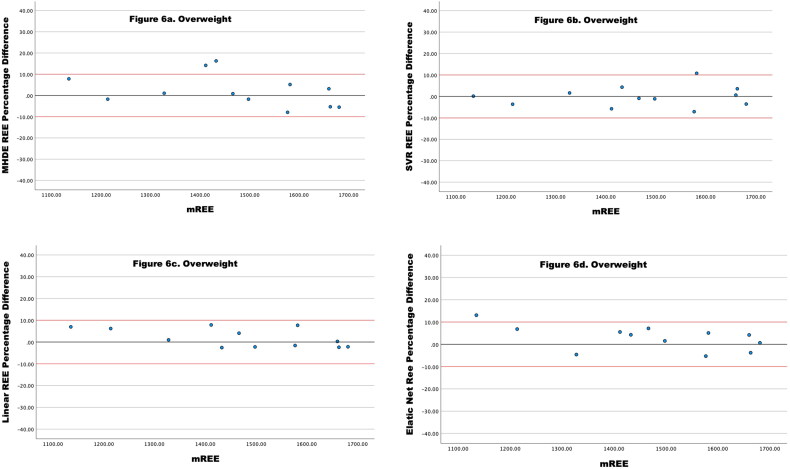
(a–d) Percentage Difference between Four different models of eREE and mREE in people receiving MHD categorized as **overweight** the black lines represent zero difference from mREE. The upper red lines represent a 10% difference from mREE. The lower red lines represent −10% difference from mR.

For participants who were overweight, accuracy was higher for the Linear Regression REE (100.0% within limits), closely followed by the SVR REE and Elastic Net; both 92.3% within limits. (Table 6 and Figure 6(b,c)) In this subgroup, the MHDE REE performed with greater accuracy than for the total group (Figure 6(a)). Again, the ML models tended to underestimate when inaccurate and the MHDE REE tended to overestimate eREE where values were out with the limits of agreement. (Figure 6(a–d))

**Figure 7.**
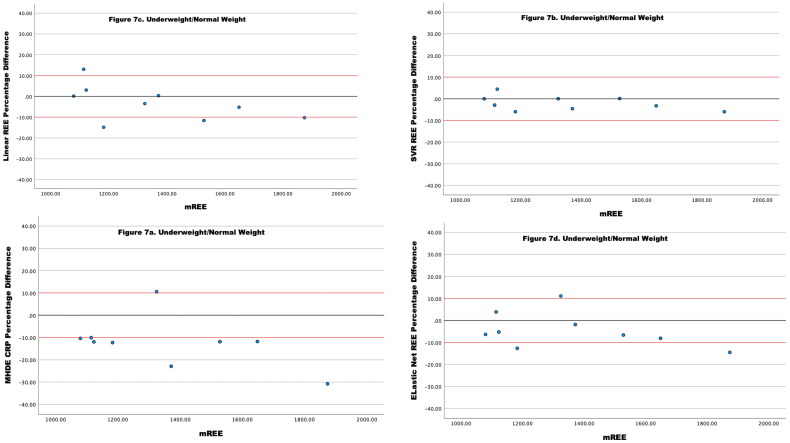
(a–d) Percentage Difference between Four different models of eREE and mREE in people receiving MHD categorized as **underweight and normal weight**. The black lines represent zero difference from mREE. The upper red lines represent a 10% difference from mREE. The lower red lines represent −10% difference from mREE.

For individuals who were of normal weight or underweight, none of the values for the MHDE REE reached the threshold for agreement and 87% of the values underestimated energy expenditure (Table 7 and Figure 7(a)). The Linear Regression REE achieved 50% of values and the Elastic Net REE achieved 62.5% of values within acceptable limits (Figure 7(c,d)). The SVR REE performed best with 100% of estimates within acceptable limits (Figure 7(b)).

**Table 7.** Features selected commonly and individually by the best three machine learning models.

Feature categories	Selected by 3 models	Selected by 2 models	Selected by 1 model
Demographic		Race	EthnicitySexAge
Anthropometric	Lean body massConicity index	Weight in kgWaist circumference in cmBia resistanceFat free mass	Height in cmBMI
Disease-Related	Length of dialysis treatmentDialysis accessDiabetes medication, insulinAnti-inflammatory medicationEtiology of CKD (hypertension)	Oral diabetes medicationAnti-lipemic medication	History of diabetesAnti-hypertensive medicationDialysis treatment timeEtiology of CKD (diabetes)
Clinical/Dynamic	Interdialytic weight gainHeart rate (bpm)	Weekly mean kt/vBody temperatureWeekly mean npcr	Weekly mean urrDystolic blood pressureSystolic blood pressure
Patient-Reported	Enjoy mealtimesWeekly appetite evaluationFeel like eatingDaily appetite rating	Intake on non-dialysis dayEnjoyment of eating on dialysis day	Difficulty following dialysis dietIntake on dialysis dayOverall appetite ratingAppetite on non-dialysis dayLack of appetite
Provider-Assessed	SGA physical examinationSGA gastrointestinal examinationSGA functional examination	SGA weight change assessmentSGA overall malnutrition rating	SGA disease assessment

bpm: beats per minute; BMI: body mass index; CKD: chronic kidney disease; kt/v: fractional urea clearance; npcr: normalized protein catabolic rate; SGA: Subjective Global Assessment; urr: urea reduction rate.

### Feature selection

**Figure 8.**
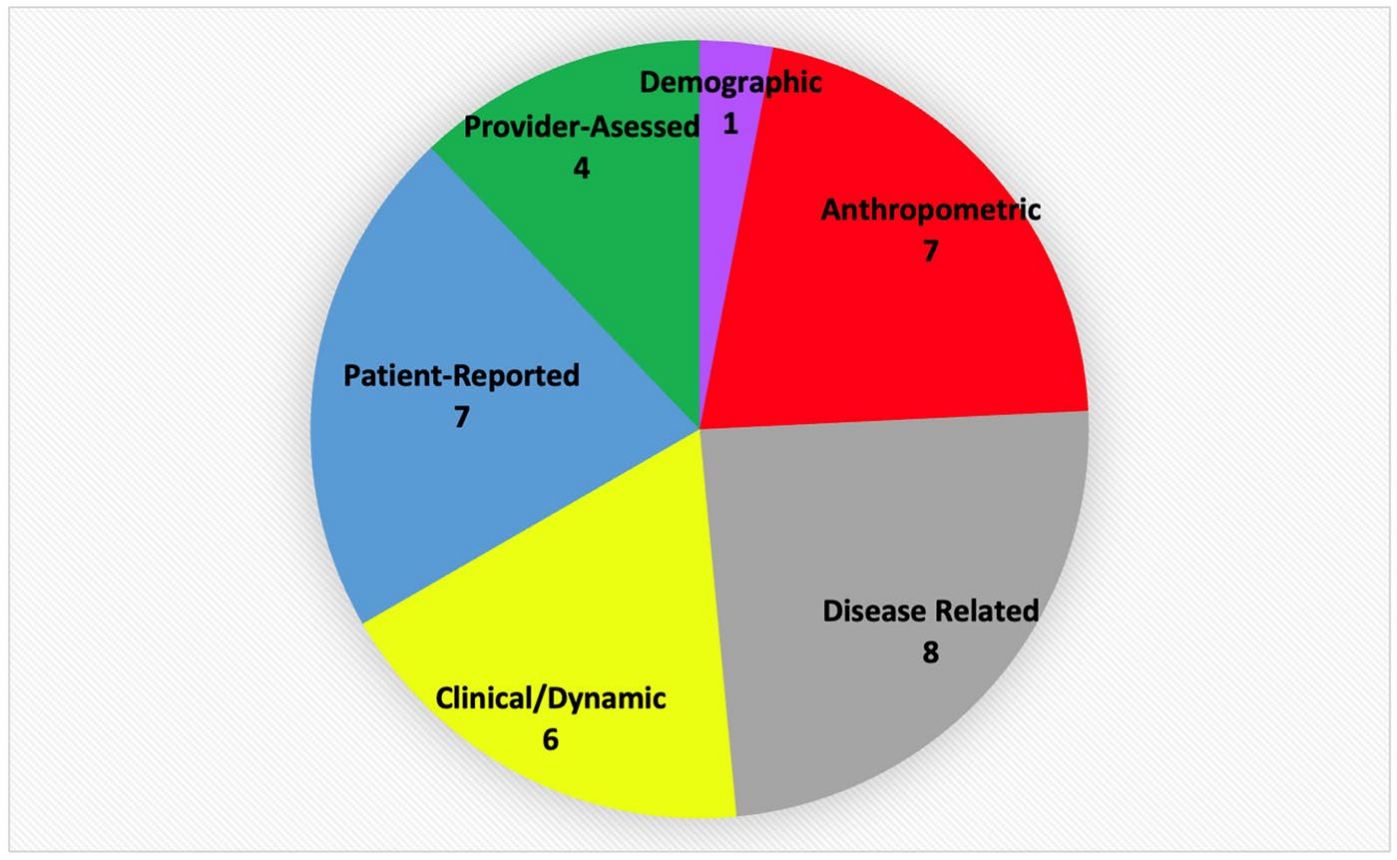
Features selected by the SVR Machine Learning model presented by the Categories: Demographic, anthropometric, disease related, clinical/dynamic, Patient-Reported and Provider-Assessed. The numerals show the actual numbers of features used.

The ML models selected 55 specific features with a very wide range of correlation to mREE (*r* = 0.77 − 0.005). In general, anthropometric features tended to show the highest association with mREE. The top 5 correlated features were lean body mass, weight in kg, dry weight 6 months previously, intradialytic weight gain and height in cm. As the modeling technique eliminated features deemed to be colinear, only two of the top features were employed by all three of the best models (lean body mass and intradialytic weight gain). The best models selected 48 features in total of which 16 were constant to all, 14 were common to two models and 18 were selected by only 1 model (Table 7). The best model (SVR) had the most individually selected features. The SVR also demonstrated the most even split of features among the defined demographic, anthropometric and clinical categories (Figure 8).

## Discussion

Our previous research demonstrated that traditional linear regression methods for predicting REE in patients with CKD are insufficiently accurate [27]. This is especially true for more vulnerable individuals as demonstrated by extremes of BMI [27]. In this pilot study, we evaluated the efficacy of ML models to better predict REE using sophisticated techniques. Although the validation sample was small, all best three models achieved improved precision over the best predictive equation in this population, the MHDE-CRP. Moreover, two of the ML models, SVR and Elastic Net, demonstrated markedly better predictive ability across the three subgroups of BMI (underweight/normal weight, overweight, obese).

### Protein energy wasting, a clinical challenge

It is estimated that up to 75% of patients treated with dialysis suffer from PEW, a unique nutritional condition that is a separate and strong risk factor for poor health sequelae and mortality [10,40]. However, identifying people with PEW can be difficult. For example, PEW can commonly occur at all levels of BMI, including in obesity, where it can be difficult for providers to pinpoint symptoms of muscle catabolism [41]. Furthermore, traditional methods for assessing malnutrition, such as the *Subjective Global Assessment* perform sub-optimally in specifically highlighting PEW in this population [40]. The ability to identify accurate energy expenditure in all individuals treated with dialysis may address diagnostic failings and provide a critical first step in selecting an appropriate care plan.

In addition to a restrictive diet, often disrupted by dialysis treatment, several factors contribute to PEW [10]. Metabolic derangements such as acidosis subdue the anabolic action of insulin and can promote the oxidation of amino acids [42,43]. Pro-inflammatory drivers from disease, dialysis treatment, access and the poor biocompatibility of dialysis methods can have a deleterious impact on appetite and directly exacerbate muscle catabolism [10,44–47]. Furthermore, hormonal changes resulting from comorbidities such as diabetes contribute to the loss of lean body mass [48]. The process of PEW is both complex and dynamic, requiring multiple strategies of nutritional, medical and lifestyle intervention [10,45–47]. As improvements in dialysis treatment (such as improved filtration and biocompatibility of medical materials) continue to evolve, dynamic methods to assess their application are increasingly appropriate [44–47] For providers to accurately assess REE, and its changes over time would provide a powerful tool in monitoring the ongoing need and effectiveness of such interventional strategies.

### Demographic features

All existing equations predicting REE incorporate age and sex, as these are highly correlated with metabolic output [15,16,20–23]. Interestingly, the best three models selected these features stochastically and neither were included by the SVR. This can be partially explained by the inclusion of LBM and FFM as variables in the dataset, which build sex into the Hume and Deurenberg Equations [49,50]. The omission of age by all but one of the best models is, however, a finding that begs further explanation. This may be consistent with our hypothesis that interactions of features are as important as strong correlations with mREE. For example, it may be that the SVR was able to establish the impact of age from its effects on other features. In our previous analysis of predictive equations from different geographical samples, we hypothesized that racial differences in model building have an impact on estimated REE [27]. In this study, both non-linear models selected race as a feature, and one also selected ethnicity.

### Disease-related features

Previous authors have found conflicting evidence regarding the impacts of clinical and disease factors on REE for dialysis patients [20–23]. Byham-Gray et al. established the importance of clinical biomarkers of inflammation (CRP) diabetes (hemoglobin A1c) and muscle catabolism (serum creatinine) in equation building [21]. Fernandes et al. also found a correlation between inflammation and mREE but did not determine that CRP explained REE variance in their sample [20]. In the best three ML models, disease-related, clinical biomarkers and vital signs constituted the largest grouping of individual features. All three of the best models selected length of dialysis treatment, type of dialysis access, diabetes medication (insulin, or oral), anti-inflammatory medication, etiology of hypertension, intradialytic weight gain and heart rate. These factors include a wide range of variables across the spectrum of CKD complications. Of note, many of the compound issues discussed in the PEW literature are represented in the list, including markers of diabetes, inflammation and type of dialysis access [10]. Indeed, it intuitively makes sense that if up to 75% of the dialysis population may be suffering from some degree of PEW, then the ML models will select PEW-relevant features in determining the metabolic drivers of REE. Our findings also agree with Ponce et al. who discovered that several disease-related, medical and dynamic factors (airway pressure, minute volume) were strong predictors in the best model for critically ill patients with AKI [28]. Although chronic CKD and critical AKI seem at opposite ends of the kidney-disease spectrum, they share the symptoms of profound metabolic alteration due to extensive medical complications.

### Patient-reported features

Another finding was the abundance of patient-reported variables related to appetite. The best models all selected ‘enjoy mealtimes’ ‘weekly appetite rating’ ‘appetite rating’ and ‘daily appetite.’ Additionally they variably selected another 8 items related to appetite, intake and mealtime enjoyment, on or after dialysis treatment. From a clinical perspective, appetite loss has long been established as a common occurrence in patients receiving dialysis, where the treatment burden leads to substantial fatigue [51]. Moreover, poor appetite is associated with elevated levels of inflammatory cytokines and is a reliable marker of the proinflammatory state [52].

### Provider-assessed features

We also observed that elements of malnutrition screening were commonly selected by the best three models, including three sections of the *Subjective Global Assessment* [53]. These included the physical examination, functional abilities and gastrointestinal symptoms. All these variables give information about an individual’s functional outputs in terms of symptoms of frailty, energy utilization and the altered faculty to eat [53]. Although the SGA has been demonstrated to be a poor diagnostic for PEW directly, it may provide valuable information as part of a more comprehensive investigation [39].

### Assessing the impact of non-linear modelling techniques

We hypothesized that by using non-linear modelling techniques, greater precision would be achieved in predicting REE across categories of BMI. The relationship of several factors influencing REE are known to be non-linear, the most fundamental being the interaction between height and weight [20–23,27,28]. In our previous research, we demonstrated that equations using linear techniques will perform with less precision at extremes of BMI as they fail to account for the changing relationship of height and weight along the correlation curve [27]. Ponce et al. observed that when ranking several ML models for the prediction of REE in patients with AKI, the non-linear models performed with greater accuracy than the linear models [28]. This study confirms these findings. Of our best three models, two were non-linear. Additionally, when the best models were assessed in subgroups of BMI, the non-linear models performed equally well for those individuals with the highest and lowest BMI. For example, the SVR REE predicted 100% of values within acceptable limits for the lowest subgroup of BMI and 85% of values within limits at the highest levels of BMI. In the past, where regression analysis has been used to create predictive equations, it has been argued that simplicity in the algorithm is a major consideration for use in clinical practice [20–23]. This has led authors to reject non-linear methods due to the difficulty of manual calculation. As almost all medical calculations are available online, or as apps on mobile devices, we argue that ‘use with a pocket calculator’ is an issue now largely redundant. Furthermore, the ability to get a large number of biomarkers from the patients’ electronic health records leads the way to easily embedding ML-based techniques for the prediction of REE.

#### Limitations of the study

The original studies in the RNKD were convenience-sampled in the Northeast and Midwest regions of the USA and hence the population was not as diverse as the national average. Additionally, those studies imposed strict medical criteria which resulted in the omission of sicker individuals. Many key variables (anthropomorphic and IC) were gathered on a non-dialysis day. This could affect a post-dialysis weight and BMI, dependent on an individual’s fluid intake and residual renal excretion. Only conventional hemodialysis was undertaken in the original studies. This gives limited insight into the clinical feature differences that may be attributable to peritoneal dialysis or more advanced techniques (such as *hemodiafiltration* or *expanded hemodialysis)*. Future research should undertake a more comprehensive review of dialysis procedures. For the purpose of this study, certain variables were omitted from the ML dataset to preserve the number of subjects available for training and validation. This includes key clinical markers such as CRP, hemoglobin A1c and serum creatinine which have been previously shown to correlate with mREE. Notwithstanding the omissions of variables, the validation set only comprised of 34 individuals, which represents a small sample size. Finally, the best model (SVR) gave substantially improved precision and a glimpse into the features that may contribute. However, the model does not generate an equation and is, therefore, less interpretable as to the direction of effect.

### Implications for practice and research

The application of a precise algorithm for predicting REE in patients receiving dialysis could present a powerful tool for providers to implement and monitor nutritional, medical and physical interventions to mitigate PEW. Current predictive equations provide inadequate precision and lack the scope to model clinical changes in metabolic needs. This is the first study to use ML to predict REE in this patient population with results that suggest a potential step change. However, this was a pilot study utilizing an existing database. To preserve the maximum sample size, some key clinical and functional biomarkers were not presented to the ML models. A research priority would be to expand the dataset to include the missing data and further explore interactions. Although our best model demonstrated high precision, it used many esoteric variables to generate accuracy. This necessarily limits the direct applicability to the clinical setting. A necessary next step is to identify the best dialysis biomarkers that could approximate the precision, and which patient-focused questions may help fill in the gaps. Thereafter the analysis should include people from different geographical locations.

ML is a process best applied to clinical spaces rich in data. A further application in the clinical nutrition field is in critical care, where data points are gathered throughout the day, precise calculation of REE is vital and REE is labile depending on the patient’s medical progress. Another related field is exercise physiology where the measured inputs of athlete nutrition, lifestyle and training schedules may shed light on the metabolic outputs of lean body mass, REE and performance.

## Conclusion

Machine learning models using non-linear techniques potentially provide a step-change in predicting REE in individuals with CKD. Feature selection by the ML models suggest that many contributing medical factors of PEW explain the variability of REE. Such information could reveal cost-effective strategies to benefit millions of people worldwide. Further research in this area is a priority.

## Data Availability

The data that support the findings of this study are available on request from the corresponding author, Laura Byham-Gray, and require a data-sharing agreement. The data are not publicly available due to restrictions, i.e. the data may contain information that could compromise the privacy of research participants.
